# Approaches and Tools to Study the Roles of Juvenile Hormones in Controlling Insect Biology

**DOI:** 10.3390/insects11120858

**Published:** 2020-12-03

**Authors:** Fernando G. Noriega, Marcela Nouzova

**Affiliations:** 1 Department of Biological Sciences and Biomolecular Science Institute, Florida International University, Miami, FL 33199, USA; Nouzovam@fiu.edu; 2 Biology Centre CAS, Institute of Parasitology, 370 05 Ceske Budejovice, Czech Republic

**Keywords:** juvenile hormone, JH titer, JH signaling

## Abstract

**Simple Summary:**

The juvenile hormones (JHs) play critical roles during insect development and reproduction. The numerous effects of JHs have generated multiple basic scientific questions, as well as prospects for the development of insecticidal endocrine disruptors. There is an increasing need for methods to identify and quantify endogenous JHs. The low titers and difficulties in working with these lipophilic compounds have often hindered the study of JH biology. In this article, we review the existing information on the detection and quantification of JH from insect samples, the development of approaches to manipulate JH titers, and the use of next-generation tools to modulate JH homeostasis.

**Abstract:**

The juvenile hormones (JHs) are a group of sesquiterpenoids synthesized by the *corpora allata*. They play critical roles during insect development and reproduction. To study processes that are controlled by JH, researchers need methods to identify and quantify endogenous JHs and tools that can be used to increase or decrease JH titers in vitro and in vivo. The lipophilic nature of JHs, coupled with the low endogenous titers, make handling and quantification challenging. JH titers in insects can easily be increased by the topical application of JH analogs, such as methoprene. On the other hand, experimentally reducing JH titers has been more difficult. New approaches to modulate JH homeostasis have been established based on advances in RNA interference and CRISPR/Cas9-based genome editing. This review will summarize current advances in: (1) the detection and quantification of JHs from insect samples; (2) approaches to manipulating JH titers; and (3) next-generation tools to modulate JH homeostasis.

## 1. Introduction

Juvenile hormones (JHs), synthesized by the minuscule *corpora allata* glands (CA), are critical regulators of insect development and reproduction. Multiple processes are controlled by JHs, including the inhibition of metamorphosis, caste determination and differentiation, stimulation of flight and migration, regulation of reproduction, control of diapause, stress resistance and immunity, as well as aging and senescence [1,2,3]. The pleiotropic effects of JH have presented a range of intriguing basic scientific questions and opportunities in an applied context as insecticidal endocrine disruptors [4,5,6]. As with other sesquiterpenoid hormones, JHs are relatively non-polar, lipophilic, insoluble in aqueous solutions and fragile, making them difficult to physically manipulate in a laboratory setting. In vivo titers generally operate in the femtomole to picomole range. The combination of low titers and challenges in physical extraction and recovery have made JHs a notoriously difficult arena for basic and applied research [7]. In this article, we review the current knowledge on the detection and quantification of JHs from insect samples, the development of approaches to manipulate JH titers, and the use of next-generation tools to modulate JH homeostasis.

## 2. Detection and Quantification of JH from Insect Samples

Seven different epoxidated JH homologs and the non-epoxidated methyl farneosate (MF) have been described, with one or more of these sesquiterpenoids detected in more than 100 insect species [3]. All these JH homologs share physical properties that hinder their isolation and quantification [7]. Biological samples containing JHs have included whole-body extracts, hemolymph, and culture media from in vitro incubated CA preparations. From these samples, JH has been quantified using bioassays, radioimmunoassays, radiochemical assays, and physicochemical assays [7]. All JH homologs share structural features, which offer advantages and disadvantages when identifying and quantifying them. Bioassays, radioimmunoassays, and radiochemical assays measure total JH activity but often do not recognize individual homologs. Physicochemical assays are the most sensitive and informative methods. Gas chromatography (GC) coupled with mass spectrometry (MS) and high-performance liquid chromatography-MS (HPLC-MS) are the best-established protocols [8,9].

**Figure 1:**
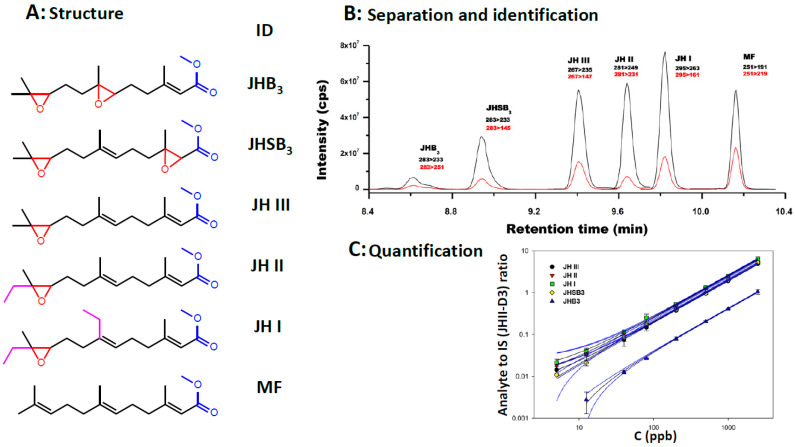
HLPC-MS/MS analysis of JH homologs. (**A**) Chemical structures of JH homologs: JHB_3_: juvenile hormone III bisepoxide. JHSB_3_: juvenile hormone III skipped bisepoxide. JH III: juvenile hormone III. JH II: juvenile hormone II. JH I: juvenile hormone I. MF: methyl farnesoate. Epoxide groups are in red. Methyl esters groups are in blue. Ethyl groups are in magenta. (**B**) HPLC separation of JH homologs: Typical LC-MS/MS peaks of JH homologs and MF. It shows the relationships between the retention times in minutes (x-axis) and the signal intensity (cps; counts per second) (y-axis). Injected mass was 125 pg (325 pg for MF and 32 pg for the internal standard –ISTD-). Retention times are in minutes. Black lines represent the signal intensity of the primary fragment used, and red lines represent the intensity of the secondary ion. (**C**) Standard curves for the quantification of JHs: relationships between the concentration of each of the five JH standards in parts per billions (ppb) (*X*-axis), and the signal intensities expressed as the ratio between the JH standard and the internal standard (IS, JH III-D_3_) (Y axis). JH III (black circle). JH II (red inverted triangle). JH I (green square), JHSB_3_ (yellow diamond) and JHB_3_ (blue triangle). The blue lines represent the 95% confidence bands, depicting the upper and lower confidence bounds for all points on a fitted line within the range of data.

The shared structural features of the JH homologs facilitated the establishment of a robust and sensitive HPLC-MS/MS (tandem MS) method. It allows the simultaneous analysis of the five most common epoxidated JHs, including JH I, JH II, JH III, JH III bisepoxide (JHB_3_) and JH III skipped bisepoxide (JHSB_3_), as well as MF [10] (Figure 1). The protocol detects JHs in the low femtomole range (pg/mL) often allowing the analysis of JH in individual insects. When an individual species of JH homolog is fragmented, it produces a unique diagnostic set of ions that enables its identification and quantification. This approach has allowed the analysis of JHs in a phylogenetically and morphologically diverse range of individual insects. This method even led to the recognition that JHSB_3_ is the original homolog that Sir Vincent B. Wigglesworth described for the kissing-bug *Rhodnius prolixus* [11] and remained unidentified since 1934 [12]. Looking forward, new developments in HPLC-MS/MS and the advent of more affordable benchtop equipment should make it even easier to quantify JHs from insect samples.

## 3. Approaches to Modulate Endogenous JH Titers

**Figure 2:**
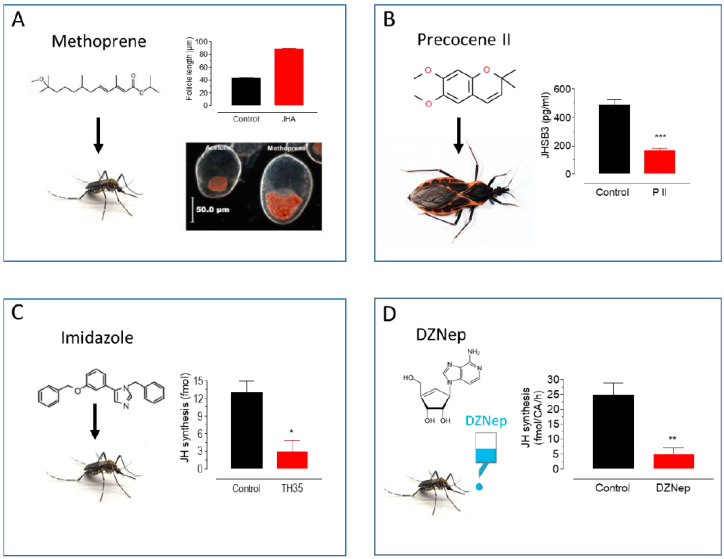
Approaches to modulate endogenous JH titers. (**A**) Methoprene topical application: Methoprene stimulates follicle development and lipid accumulation in *Ae. aegypti* females [20]. (**B**) Precocene II topical application: P II decreases JHSB_3_ titers in *Dipetalogaster maxima* [35]. (**C**) Imidazole treatment: Imidazoles inhibit JH synthesis in *Ae. aegypti* females [30]. (**D**) DZNep in vivo treatment: DZNep added to the sugar inhibits JH synthesis in *Ae. aegypti* females [34]. * *p* < 0.05, ** *p* < 0.01, *** *p* < 0.001.

JH titers are finely controlled, and alterations of JH homeostasis can interfere with a wide variety of biological functions including development, molting, and reproduction. Insect growth regulators (IGRs) are natural or synthetic compounds that interfere with JH homeostasis [13]. These compounds either decrease JH biosynthesis and transport, increase JH catabolism, or interfere with other elements of the JH signaling pathway [5,6]. In addition, JH analogs (JHA), such as methoprene, fenoxycarb, and pyriproxyfen, are functional mimics of the endogenous JHs that increase JH signaling, preventing metamorphosis [14] or interfering with normal reproduction [6,15] (Figure 2A). JHs act as an agonist by binding to the JH receptor [16,17]. This methoprene-tolerant (Met) protein is conserved in all insects that have been investigated and has a paralog in *Drosophila melanogaster* (Gce) [18]. Met is an intracellular receptor in the basic HLH (helix–loop–helix)—(bHLH–PAS) family of transcriptional regulators. All known JH homologs and JHAs operate by interacting with this unique Met protein [17,18].

In addition to their utility as insecticides, synthetic JH mimics are essential research tools to study the role of JH signaling in vivo and in vitro systems [6]. For example, JH signaling can be easily increased with the topical application of methoprene [19,20]. The JH analogs are more stable than natural JHs, and are highly active in a broader spectrum of insects. This feature is useful when trying to assess whether JH signaling is important in controlling the physiological process under study. On the other hand, the relative molecular stability of JH analogs becomes a problem when studying processes that require only temporary increases in JH titer; for instance, treating mosquito or *Drosophila* larvae with JHA results in lethality, preventing pupae from reaching adulthood [21,22].

In contrast, deliberately reducing JH titers has proven more difficult. However, some efficient anti-juvenile hormones compounds have been described. Precocenes are strong inhibitors of CA activity (anti-allatotropins) in several species of insects [23,24]. Precocenes have anti-JH effects by causing necrosis of the CA (pro-allatocidins) [25] (Figure 2B). An alternative approach is to modulate hormonal catabolism. JH degradation by JH esterase (JHE) plays an important role in lowering JH titers [1]. JHE over-expression in early larval instars has been shown to cause precocious metamorphosis through the elimination of JH [26]. In addition, recombinant JHE (rJHE), injected in vivo, has been employed as an anti-JH agent in several insects [27,28,29]. Alternatively, imidazole-containing compounds are potent anti-JH compounds [30]. They effectively inhibited JH biosynthesis by the CA of the cockroach *Diploptera punctata*. Imidazoles were also effective inhibitors of purified cockroach epoxidase (CYP15A1) in the conversion of MF to JH III [31]. Imidazoles are also strong inhibitors of JH synthesis in Diptera as well [32] (Figure 2C).

The modulation of JH biosynthesis can also modify JH titers. A critical enzyme in JH synthesis is juvenile hormone acid methyltransferase (JHAMT) [33]. An inhibitor of JHAMT is 3-deazaneplanocin A (DZNep), which inhibits JH synthesis in vitro by the CA of female adult *Aedes aegypti* in a dose-response fashion [34]. In vivo experiments, with the addition of DZNep to the sugar ingested by mosquitoes, also resulted in a dose-response decrease in JH synthesis and JH hemolymph titers (Figure 2D), as well as a decrease in the expression of early trypsin, a JH-dependent gene. These results suggest that DZNep can lower JH synthesis and titer in experiments evaluating JH-controlled processes in insects.

## 4. Next-Generation Tools to Modulate JH Homeostasis

**Figure 3:**
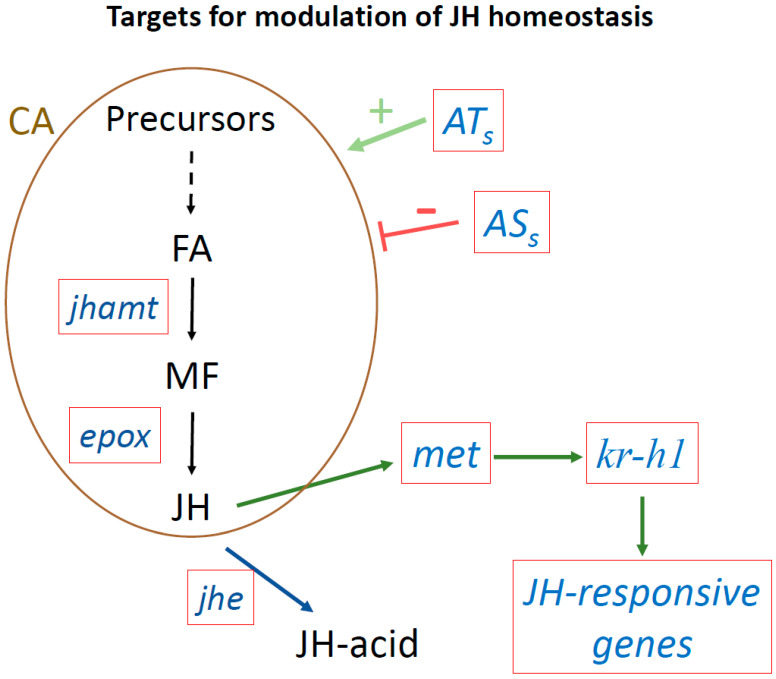
Targets to modulate JH homeostasis using next generation tools. Targeting genes playing critical roles modulating JH titers or JH signaling. Red boxes show target genes. Juvenile acid methyl transferase (*jhamt*) transforms farnesoic acid (FA) into methyl farneosate (MF). Epoxidase (*epox*) converts MF into JH. JH esterase (*jhe*) converts JH into JH-acid. The *met* gene encodes the JH receptor. *Krüppel-homolog 1* (*Kr-h1*) is as an early inducible gene in the JH signaling pathway, that activates the transcription of many JH-inducible genes. ATs, allatotropins that stimulate JH biosynthesis; ASs, allatostatins that inhibit JH synthesis; CA, *corpora allata*.

Changes in JH homeostasis can be generated by modulating the expression of many different critical genes involved in diverse aspects of JH biology (Figure 3). Reducing gene expression can be accomplished with RNA interference (RNAi), while enhancing gene expression can be done by driving ectopic genes with tissue-specific promoters in a transgenic context. Gene editing has become a highly efficient method to alter insect genomes. It can be used to either express genes with known JH-modulatory effects or to knock out genes playing critical roles in modulating JH titers or in JH signaling pathways.

In insects, RNAi generally causes loss-of-function phenotypes by the degradation of specific transcripts, though it may also exert translational inhibition. It is an efficient and simple reverse functional genomics approach widely used to study the actions of insect genes [36]. This gene knock-down technique allows insect’s loss-of-function (LOF) analyses without creating null mutants. Novel RNAi delivery systems, including ingestion, injection, cuticular penetration, delivery by microbes, and the use of nanoparticles, are facilitating the use of RNAi in insect’s research and control [37]. The RNAi effect acts systemically or with tissue-specificity if driven by transgenes [38,39]. RNAi has been a valuable technique in different areas of JH research. RNAi knock-down of Met in the beetle *Tribolium castaneum* produced precocious metamorphosis phenotypes consistent with disrupted JH signaling, thus helping to establish the identity of the JH receptor [40]. Later, the study of *Drosophila* Met-Gce double mutants provided conclusive genetic evidence that definitively confirmed the JH receptor role of Met-Gce [18]. A yeast-derived Gal4-UAS system, achieving temporal and tissue specific control, was used to validate that ecdysis-triggering hormone (ETH) functions as an allatotropin, inducing JH synthesis by the CA of *Drosophila* [38,39]. RNAi constructs targeting the ETH receptor gene (*ethr*) were introduced into the genome using the CA-specific driver *jhamt*-GAL4, resulting in a marked reduction (>70%) of JH levels [38]. Meanwhile, cell-specific Gal4 driver-mediated expression of pro-apoptotic genes in Inka cells (the cells that produce ecdysis triggering hormone) also resulted in a significant decrease in JH signaling [38,39].

*Krüppel-homolog 1* (*Kr-h1*) is an early inducible gene in the JH signaling pathway downstream of the Met receptor in insects [41]. RNAi has been successfully used to validate the roles of the Met receptor and Kr-h1 in JH signaling in *R. prolixus*. Premature depletion of Kr-h1 [42] or Met [43] triggered precocious adult development. Modulation of Kr-h1 expression by RNAi has often been used to investigate JH/Met-mediated control of gene activity [44]. Moreover, the study of the expression of JH-regulated genes is a reliable and straightforward approach to validate the putative role of JH in modulating physiological or behavioral processes. In mosquitoes, the early trypsin mRNA, a well-known JH-dependent gene [45], has been widely used as a model for JH signaling studies [46], as well as a marker for JH titers. A study has described an early-trypsin-GAL4 > UAS-enhanced green fluorescent protein (EGFP) system to monitor JH signaling [47]. The system revealed that in early-trypsin GAL4 > UAS-EGFP female mosquitoes, the intensity of the midgut-specific EGFP signal was significantly and strongly correlated to the early trypsin gene transcript levels, as well the JH titer; probing the usefulness of using the expression of this gene as a marker for JH-signaling activation [47].

In many insect systems, RNAi does not always mediate efficient gene knock-down, and sometimes mRNA levels are reduced by less than 75% [48]. Residual gene expression can mask phenotypes in RNAi experiments, which complete loss-of function alleles induced by mutagenesis might uncover. In this context, CRISPR/Cas9-based editing approaches offer a more clearly defined tool for manipulating gene expression in insects [49]. Highly efficient and specific knockout of genes can be achieved by injecting embryos with an in vitro-synthesized single guide RNA (sgRNA) and Cas9 mRNA or protein [50,51].

In *Drosophila*, precise temporal-spatial activation of the expression of transgenes that modulate JH titers or signaling can be achieved using binary CRISPR/Cas9 systems. These systems utilize a driver line to promote high-level, tissue- or developmental stage-specific expression of the “JH-modulatory” gene in a responder line. Spatial and temporal control of gene manipulation in *Drosophila* has been achieved via drug-activated Cas9 nucleases [52]. Drug-inducible CRISPR/Cas9 systems permit the study of genes at later stages where early lethality is a problem; combined with tissue-specific expression of Cas9 or sgRNAs, it results in spatiotemporal control [52]. Genome editing with “first-generation methods” such as TALENs has been successfully employed to knock out genes playing critical roles modulating JH titers or JH signaling. For example, this method was used to produce a knockout mutant of the rate-limiting JH acid methyl transferase (*jhamt*) and the two JH receptor genes (Met1 and Met2) in the silkworm *Bombyx mori* [53]. Experiments with these *Bombyx* null mutants revealed that the larval status can be maintained by a JH independent mechanism in very early larval instars [53]. CRISPR/Cas9- generated null mutants of juvenile hormone esterase showed extended larval growth in *Bombyx* [54]. Similarly, the knockout of *jhamt* resulted in a significant reduction in fecundity in *Drosophila* [55]. Using CRISPR/Cas9 gene editing, a study with *Ae. aegypti* mosquitoes showed that silencing the JH receptor (Met) has effects similar to those attributable to a drop in JH titer [56]. The functions of the juvenile hormone binding protein (mJHBP) have been studied in an mJHBP-deficient null mutant *Ae. aegypti* mosquito; the studies revealed that JH functions in mosquito immunity and hemocyte development in a manner that is perhaps independent of canonical JH signaling [57].

## 5. Conclusions

The study of different aspects of JH biology has been traditionally hampered by the difficulty of working with this lipophilic hormone, elusive in low physiological concentrations. In this review, we summarized some of the traditional and novel tools to study the roles of JH in controlling insect biology. Mass-spectrometry methods represent the gold standard for the detection and quantification of JH from insect samples, providing fast, simple, accurate, ultra-trace quantitation of all major JH homologs. The ability to modulate endogenous JH titers, accomplished using JH analogs or JH-synthesis modifying drugs, now can be more efficiently attained using next-generation strategies. These, with their potential for precise temporal- and tissue-specific control, can modulate the expression of many different critical genes involved in diverse aspects of JH biology.
